# Hospitals early challenges and interventions combatting COVID-19 in the Eastern Mediterranean Region

**DOI:** 10.1371/journal.pone.0268386

**Published:** 2022-06-03

**Authors:** Hamid Ravaghi, Vanessa Naidoo, Awad Mataria, Merette Khalil

**Affiliations:** 1 Universal Health Coverage and Health Systems Department, World Health Organization, Eastern Mediterranean Regional Office, Cairo, Egypt; 2 Division of Emergency Medicine at the University of Cape Town, Cape Town, South Africa; Maragheh University of Medical Sciences, ISLAMIC REPUBLIC OF IRAN

## Abstract

**Background:**

During rapidly evolving outbreaks, health services and essential medical care are interrupted as facilities have become overwhelmed responding to COVID-19. In the Eastern Mediterranean Region (EMR), more than half of countries are affected by emergencies, hospitals face complex challenges as they respond to humanitarian crises, maintain essential services, and fight the pandemic. While hospitals in the EMR have adapted to combat COVID-19, evidence-based and context-specific recommendations are needed to guide policymakers and hospital managers on best practices to strengthen hospitals’ readiness, limit the impact of the pandemic, and create lasting hospital sector improvements towards recovery and resilience.

**Aim:**

Guided by the WHO/EMR’s “Hospital readiness checklist for COVID-19”, this study presents the experiences of EMR hospitals in combatting COVID-19 across the 22 EMR countries, including their challenges and interventions across the checklist domains, to inform improvements to pandemic preparedness, response, policy, and practice.

**Methods:**

To collect in-depth and comprehensive information on hospital experiences, qualitative and descriptive quantitative data was collected between May-October 2020. To increase breadth of responses, this comprehensive qualitative study triangulated findings from a regional literature review with the findings of an open-ended online survey (n = 139), and virtual in-depth key informant interviews with 46 policymakers and hospital managers from 18 out of 22 EMR countries. Purposeful sampling supported by snowballing was used and continued until reaching data saturation, measures were taken to increase the trustworthiness of the results. Led by the checklist domains, qualitative data was thematically analyzed using MAXQDA.

**Findings:**

Hospitals faced continuously changing challenges and needed to adapt to maintain operations and provide essential services. This thematic analysis revealed major themes for the challenges and interventions utilized by hospitals for each of hospital readiness domains: Preparedness, Leadership, Operational support, logistics, supply management, Communications and Information, Human Resources, Continuity of Essential Services and Surge Capacity, Rapid Identification and Diagnosis, Isolation and Case Management, and Infection, Prevention and Control.

**Conclusion:**

Hospitals are the backbone of COVID-19 response, and their resilience is essential for achieving universal health coverage. Multi-pronged (across each of the hospitals readiness domains) and multi-level policies are required to strengthen hospitals resilience and prepare health systems for future outbreaks and shocks.

## Background

Achieving universal health coverage (UHC) and protecting global health security (GHS) are central to achieving the right to health for all and the Sustainable Development Goals. The blueprint lies within strengthening health systems and hospitals in their recovery from this global pandemic [1–4].

Originating in Wuhan, China at the end of 2019, COVID-19 is the third coronavirus infection in two decades, after severe acute respiratory syndrome (SARS) and Middle East respiratory syndrome (MERS) and was declared a global pandemic by March 2020 affecting every country worldwide with increasing cases and fatalities despite vaccination efforts [3, 5, 6]. Perceived risk of acquiring disease has led many governments to institute a variety of infection control measures, including but not limited: restrictions to travel, enforcing national curfews and lockdowns, shutting of airports, schools, malls, and public spaces, limiting mass gatherings, issuing laws to implement preventative measures of social distancing and weaking masks among others [3, 7, 8]. However, across numerous low-and-middle-income-countries (LMICs), including those in the Eastern Mediterranean Region (EMR), various challenges including the absence of testing, critical care capacity, climate, war, distrust, and large refugee populations complicate implementation of these measures and threaten proactive outbreak response [4, 9].

The EMR comprises of 22 countries, ranging from Morocco to Pakistan, with extreme differences in wealth, political stability, health system resilience, and emergency preparedness [10]. Karamouzian et al. described the disparities in the EMR, where some of the wealthiest oil-producing countries globally are contracted by a increasing extreme poverty rate and more than 20 million people are living on less than US$1.9 per day [11]. In the Region, 11/22 countries are graded emergencies, it’s home to 43% of those who need humanitarian assistance globally, and the source of 64% of the world’s refugees [12]. In efforts of leaving no one behind, impetus is placed on protecting vulnerable populations and those with increased health risks due to their intersectional identities and social determinants [13, 14]. Millions of families lacked or could not afford health care without suffering financial hardships; with these vulnerabilities exacerbated in fragile and conflict affected settings (FCS) [10].

The first case of COVID-19 in the EMR was reported in Iran in late February 2020, and by early May, cases were reported across all 22 countries of the EMR [11]. To date, two years later, there are approximately 20 million cases with almost 325,429 deaths in the EMR [12]. Hospitals and health systems in the Region faced unprecedented pressures of combatting this outbreak, providing essential services, and responding to shocks and humanitarian emergencies. In August 2020, about 75% of essential health services were disrupted (in 13/22 EMR countries), affecting routine immunizations, dental services, rehabilitation services, and family planning, and further exposing the fragility of medical supply chains [15, 16]. In the EMR, the vast majority (around 80%) of hospital beds are in the public sector, ranging 3.9 hospital beds per 10,000 population in Afghanistan to 32 beds per 10,000 in Libya [17]. Prior to the pandemic, hospitals across the Region faced numerous challenges, whether related to financial, material, or human resources, inaccessible or ineffective services, or poor quality [18]. During rapidly evolving outbreaks, the provision of care by hospitals and health care facilities tends to become interrupted, and in this case, hospitals have become overwhelmed in responding to COVID-19 surges with additional challenges often exacerbated by humanitarian and graded emergencies [18].

Global health experts highlighted the importance of strengthening and mobilizing hospitals and health systems for outbreak response to protect GHS and ensure patient’s rights to health; the proactive and systematic implementation of preparedness and response plan is therefore essential to improve and facilitate management within hospitals [19–28]. Studies on hospital preparedness for biological outbreaks confirmed the need for hospitals to strengthen their capacities in planning, surge capacity, communication, training and education, medical management, surveillance, and standard operation process, among others [29]. Hospital preparedness efforts must focus not only on infrastructure, surge capacity, and supplies, but also on staff, including protection from nosocomial transmission and promotion of mental wellbeing [5]. Yet, across the Region, a lack of contingency planning and insufficient availability of resources threaten hospitals readiness to respond to outbreaks [30]. Many hospitals normally operate at near-surge capacity, without established and functional referral pathways, and even a small rise in patient numbers during an emergency can pressure hospitals to work beyond their functional capabilities [31]. While hospitals around the world have adapted to respond to the pandemic, evidence on responses and interventions from resource-restrained contexts as the EMR is limited [32]. Given the complex challenges faced by hospitals in the EMR, there is a need for evidence-based and context-specific recommendations to guide policymakers and hospital managers (HMs) on best practices to strengthen hospitals’ readiness for various types of hazards and emergencies, limit the impact of the COVID-19 pandemic on local health systems, and create lasting hospital sector improvements towards recovery and resilience.

This study presents the experiences of EMR hospitals combatting COVID-19 between March-Oct 2020, including their challenges and interventions across major hospitals’ readiness domains, to inform improvements to pandemic response, policy, and practice.

## Methods

**Fig 1:**
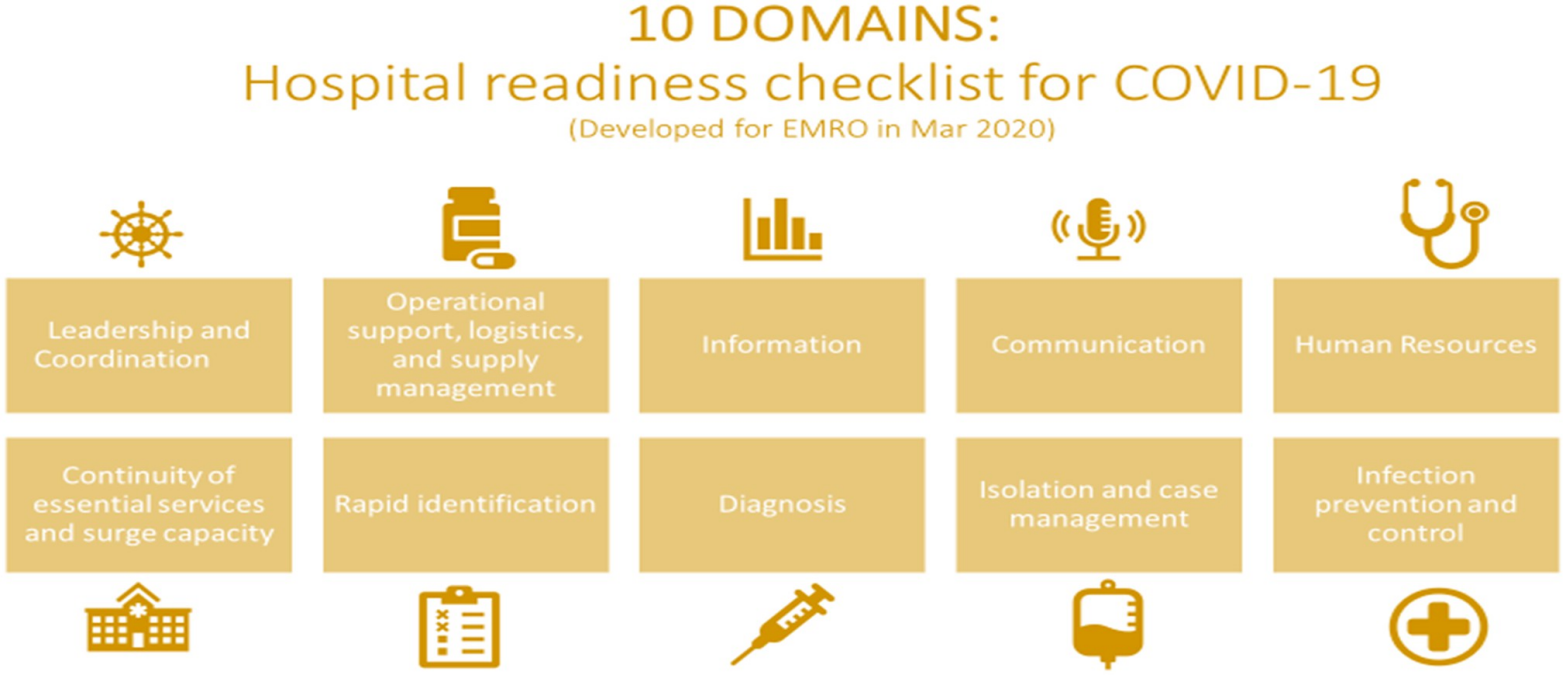
Hospitals’ readiness checklist for COVID-19 (WHO/EMRO, 2020).

This study triangulated the findings from a literature review, online survey, and key informant interviews (KIIs), to comprehensively capture hospitals’ diverse and complex experiences in combatting COVID-19 from the Region. The ***“Hospital Readiness Checklist for COVID-19”*** (Fig 1) was developed by EMRO served as the guiding analytical framework for this study, informed the development of the study tools (interview guide, codes list, and survey tool), and was used to organize the findings [33]. This study received ethical approval from the Regional Ethical Review Committee of the World Health Organization’s Eastern Mediterranean Regional Office, which permits research to be conducted in the 22 countries of the Region.

### Data collection

Both qualitative and quantitative data were collected for this study. The findings are based mostly on qualitative data, which was collected from three sources, including a comprehensive scoping literature review, in-depth interviews with key informants, and five open-ended questions from the online survey. Simple descriptive quantitative data was collected using the survey tool to capture the most faced challenges and most frequently utilized checklist interventions across a sample of hospitals in the EMR. All study tools were reviewed by regional health systems experts, piloted, and modified accordingly. Ethical considerations were applied during KIIs and the online survey to protect participant confidentiality.

### Key informant interviews

To deeply understand the complex, diverse, and context-specific hospitals’ experiences in the EMR, semi-structured key informant interviews were conducted between July and October, 2020 (S1 Appendix). KIs were recruited by respective WHO country offices, 18/22 countries provided nominations, and 46 KIIs were interviewed online (using Zoom) for 50–90 minutes by 2 members of the research team. To optimize diversity, comparability, and transferability of findings, no restrictions were placed on type or size of facility, participants represented all but 3 countries in the EMR, ranging from low, middle, and high-income countries, including countries in conflict settings and emergencies, and included professionals across various disciplines. The selection criteria for KIs was based on their role as policymakers, hospital managers, and/or members of senior management teams in hospitals treating COVID-19. Participation was voluntary and participants provided their informed verbal and written consent after receiving all relevant information introducing the project, a detailed consent form, and a copy of the topic guide. Most participants accepted the invitation for interviews, only in a few cases, participants were not able to participate due to high pressures and demanding schedules faced by hospital managers and front-liners during the pandemic. In these cases, KIs nominated other relevant stakeholders which were interviewed in their stead.

A topic guide of semi-structured questions was created based on the guiding framework to capture the journey of hospitals preparedness from the first months of the response (including challenges and interventions) compared to the later months. Interviews were conducted mostly in English, with few conducted in either fully or partially in Arabic, Persian, or French. To increase data validity and reliability, active listening, and probes along with prolonged engagement and immersion with the data were used. To improve the confirmability of results, interviews were transcribed using an electronic software and reviewed by all members of the research team and cross-referenced against the notes taken by the interviewers. In non-English KIIs, a translation was made by the research team, and main notes shared in English for summary, discussion, and consensus. A record of analytical activities was also kept. The interviews were audio-recorded and kept in secure files to be deleted within 2 years of project finalization. To improve credibility and transferability, the initial findings were shared with participants for discussion and feedback. The results were presented in two webinars with key informants and technical experts, each with over 100 participants. The feedback was positive and did not significantly change the results.

### Online survey

An online questionnaire using GoogleForms was developed, piloted, and disseminated widely. Hospital managers (HMs), clinical directors, management teams, senior front-line health professionals were invited to participate, and the link was shared through WHO country offices to key national stakeholders, their staff and professional networks via email and social media platforms such as Whatsapp. All key informants received a link to the survey, some of which completed the survey while others shared it among their respective networks. To access the survey, respondents read a brief introduction to study objectives and overview of ethical considerations; all responses were collected anonymously and voluntarily. The survey consisted of four sections: 1) participant demographics, 2) hospital demographics, 3) simple data type questions on hospital responses to COVID-19 (related to the challenges and interventions across the various checklist domains) and 4) open-ended questions regarding experiences, challenges, lessons learned, and the roles and expectations of hospitals, governments, and WHO which provided rich qualitative data for further analysis and triangulation. To optimize responsivity, survey data was collected between July and October 2020, and follow-up message were sent regularly to remind participants to respond and widely share the survey.

### Literature review

**Fig 2:**
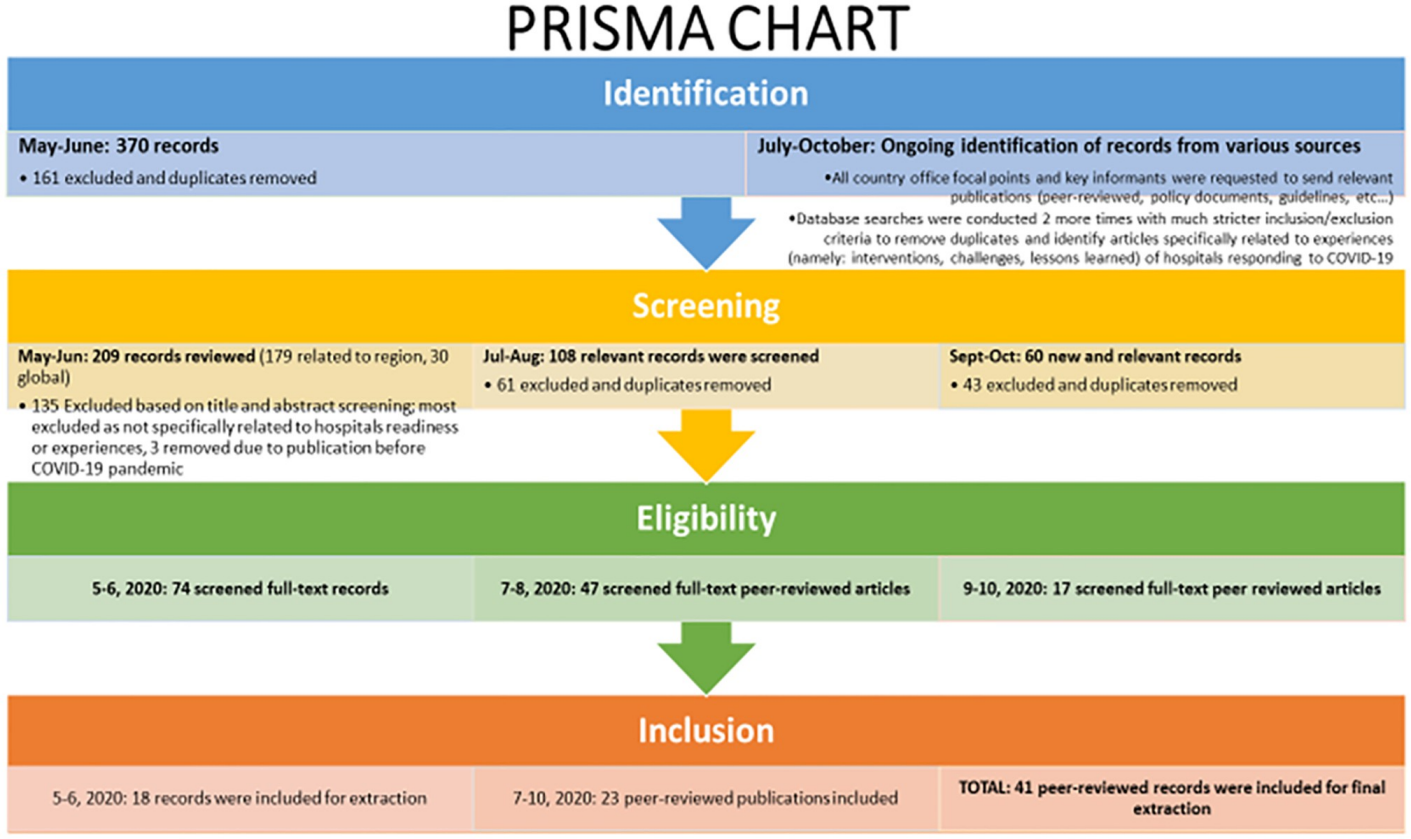
PRISMA chart for literature review and articles inclusion.

Challenged by the dearth of regional literature specifically on hospitals early responses to the pandemic, as well as the evolving nature of the virus and evidence, A scoping literature review was also conducted to answer the research question: “What were hospitals’ experiences combatting COVID-19 in the EMR?” To ensure that all new and relevant publications were identified and screened for eligibility and inclusion, literature was searched three times between May-October 2020 (May-June, July-August, September-October 2020). Major databases and journals searched were PubMed, CINAHL, Google Scholar, BMJ and BMC, SpringerLink, and the EMHJ using a combination of keywords and their variations including but not limited to: ‘Hospital’, ‘response’, ‘COVID-19’, each of the 22 EMR countries, and each of the 10 checklist domains. The MeSH terms used included: ((("Hospitals"[Mesh]) AND ("COVID-19"[Mesh] OR "SARS-CoV-2"[Mesh])) AND ("Middle East"[Mesh] OR "Africa, Northern"[Mesh]))), further details on the search strategy can be found in Fig 2. Searches were limited to articles on humans, published starting 2020, written in Arabic, English, and French (with most publications written in English). A review of reference lists and snowballing were conducted to identify other relevant literature. All entries that were not specifically related to the hospitals’ challenges, interventions, or lessons learned in responding to COVID-19 in the 22 countries of the EMR were excluded. A total of 41 peer-reviewed publications were included for full-text revision and extraction during the study period (Fig 2). To remain updated with recent literature at the time of publication, the same search strategy was applied between Jan-Jun 2021, yielding 67 entries of which 12 were included. This study also reviewed and analyzed over 100 pieces of grey literature including ministerial or national response plans, strategies, presentations, WHO and UN partner reports and other relevant publications by key partners in hospitals management.

### Data analysis

Inspired by Braun and Clark, a thematic analysis was used, using the 10 domains of WHO’s Hospitals Readiness Checklist as the guiding framework [34]. Qualitative data from the three sources [interviews, open-ended survey responses, and documents analysis) was thematically analyzed using both deductive and inductive methodologies: an analytical code list was developed deductively with codes and sub-codes of the interventions proposed for each of the 10 checklist domains (Fig 1), while a detailed inductive approach was used in reviewing and analyzing the interviews to identify additional themes that may have been missed. The qualitative data was coded using MaxQDA2020. Guided by the domains of the checklist, open coding was used and themes were organized and merged accordingly. Two coders discussed the completeness of the data and reached consensus regarding data saturation when no new themes emerged. A few additional interviews were conducted to confirm data saturation. Following discussions, the coded segments were sorted to identify the main challenges, interventions, and lessons learned for each checklist domain. In addition to the checklist domains, the theme of preparedness was added due to its frequency. The findings were triangulated with survey results and a literature review for further validation.

Following data cleaning, a total of 139 relevant and complete survey responses from 14/22 EMR countries were reviewed and a simple descriptive analysis was conducted using Microsoft Excel. Almost equal numbers of males and females responded to this online survey, almost 60% were between 35–50 years of age, and 64% of respondents had over 10 years of experience working in hospitals. About 55% of participants were senior members of hospital management teams and 8% were policymakers. About 60% of participants worked in tertiary hospitals, while 88% indicated that their hospital was government or publicly owned and operated. About 93% of survey responses indicated that their hospital had admitted a COVID-19 patient while 75% indicated that their hospital was designated for COVID-19 response.

## Findings

### Preparedness

The first case of COVID-19 globally was announced December 31, 2019 within a month it was announced as a Public Health Emergency of International Concern (PHEIC) yet many KIs from many EMR countries reported not starting preparedness and response plans until a case was diagnosed in their borders. The delays in planning and implementing national preparedness measures in many EMR countries resulted in a disorganized and inefficient management of the response with implications on the hospital sector [35–37].

In FCS, KIs noted that fragmentation and inadequate implementation of preparedness plans were the result of some health system characteristics such as: chronic underfunding in health, high out of pocket expenditures, competition from the private sector, understaffing in public hospitals, poor referral pathways, weak IPC and emergency infrastructures (nationally and at hospital-level). KIs noted that the lack of a holistic or systems-approach to preparedness and the presence of two or more governments in some FCS resulted in conflicting guidelines and plans between opposing parties and levels of government, duplication of tasks, inefficient allocation of resources (human, material, and financial).

***“Because of the fragmentation*, *there is overlap and duplication of tasks; more than one side is doing procurement; the two parties are challenging each other*, *each has different strategies for the taskforces*, *they are developing conflicting policies.”***

***(KI-22)***

These early challenges were attributed to fragmented coordination, poor testing mechanisms, weak quarantine measures, and inadequate infection control for travelers and returnees before lockdowns [35, 38–41]. KIs in Afghanistan, Iraq, Libya, Palestine, Somalia, Sudan, and Syria confirmed these national and health systems challenges contributed to inefficiencies in hospitals’ responses. Moreover, many hospitals in the Region were not sufficiently prepared to respond to this pandemic. In some countries, hospitals’ emergency preparedness was tailored for surges due to humanitarian emergencies, natural disasters, and mass casualties without sufficient consideration for infectious disease outbreaks. In others, the lack of preparedness plans and protocols at facility level impeded prompt implementation; some hospitals applied generic national protocols related to surge and IPC, which were not specific to facility-level operations [42]. In countries where hospital-level plans existed, KIs from LMICs frequently reported that these plans were mainly on paper without adequate training or simulation exercises to evaluate their implementation and impact. Further, many hospitals in the Region were underprepared for surges from cross-border infections, and infections from community spreading following religious holidays; this continues to threaten the response in subsequent waves.

To overcome these challenges, at national and facility level, multi-sectoral and multi-specialty emergency operating centers (EOCs) and COVID-19 management teams were established. At facility-level, the establishment of EOCs was reported by 74% of survey participants, most utilizing multidisciplinary committees to lead and evaluate the response. These multi-disciplinary teams contributed to proactive planning for anticipated surges around holidays, evaluation of lessons from previous MERS, SARS, multi-drug resistant infectious disease outbreaks, rapid decision-making in resource allocation, policy development, and updating standard procedures and clinical guidelines. In some FCS with humanitarian operations, country preparedness and response plans (CPRPs) served as a basis to guide the coordination of the emergency response including the health and hospital sectors [43–46].

***“Our hospital established a coronavirus response committee*, *which was activated when the first case was diagnosed by mid-April 2020*, *comprising the CEO*, *COO*, *and the Medical Directors*, *the infectious disease specialists*, *pharmacy/supply services*, *front-liners*, *and support services like cleaners.”***

***(KI-19)***

The interlinkages between facility and policy levels were prominently described as hospital directors and members of hospitals management teams provided technical expertise and advised national response committees. Additionally, health systems and hospitals readiness assessments were conducted to evaluate preparedness; 4 countries in the Region utilized or adapted the hospitals readiness checklist to evaluate preparedness.

Hospitals in the EMR applied various interventions to improve, implement, and practice preparedness including but not limited to early procurement of supplies, increasing staffing for surges, issuing hospital wide alerts or codes, running drills and simulations exercises, and conducting daily meetings and epidemiological briefings to evaluate risks, actions, and responses. HMs also raised the importance of adaptability, flexibility, and agility in altering hospital infrastructure to create new spaces to treat and triage patients with physical distancing (such as expanding ventilated ICU rooms for critical patients) and establishing zones for adequate IPC measures (such as donning and doffing) for better and safer patient flows. HMs confirmed attempts for continuously updating and adapting the guidelines and protocols to ensure efficient and safe care in line with the latest and evolving evidence-based recommendations [30, 47]. KIs from a third of the Region further reported that accredited hospitals and those implementing Patient Safety Friendly Hospital Initiatives (PSFHI) were more prepared that their counterparts.

***“Accredited hospitals were a resource for the non-accredited hospitals*, *private and governmental hospitals*, *leading by example by setting up screening areas*, *triage systems*, *protocols for critical care and infection control.”***

***(KI-9)***

### Leadership and coordination

Across the EMR, especially in FCS, the politicization of COVID-19 and centralization of the early response in the capital cities and in designated-COVID-19 public facilities quickly resulted in hospitals being overwhelmed. KIs particularly the Region’s LMICs, attributed disrupted procurement, inconsistent protocols, inequitable resource distribution, shortages of supplies and equipment, violence against health workers, and fragmented and duplicated policies and operations, to inadequate leadership at national and facility levels. KIs from numerous LMICs, especially in FCS, contributed the weak response to the lack of representation and engagement of stakeholders and public health experts. This disconnection from the situation on the ground resulted in inefficient planning, poor enforcement of protocols, guidelines, and other preventive measures which exacerbated the growing distrust in governmental authorities and hospitals; this is still threatens health systems responses in the face of subsequent waves. HMs expressed that their limited autonomy and, in some cases, limited managerial capacities resulted in weak implementation of basic IPC or triage guidelines, delayed procurement of critical supplies, and inadequate recruitment and staffing for COVID-19 response. Moreso, KIs in LMICs and FCS further confirmed their reliance on central government for procurement of supplies, HR management (including recruitment and staffing for surges), and response plans and guidelines:

***“Hospitals don’t have the inability to procure anything*, *the state government company is responsible for procuring medicines and supplies and [controls everything]*. *Last week [08/2020]*, *people [including health workers] had a strike against the government because patients died for lack of oxygen*, *and this is a chronic problem*, *across all health facilities there are shortages and none of the hospitals have the authority to procure it.”***

***(KI-5)***

KIs further attributed the untimely and ineffective response to decentralization without adequate coordination of stakeholders, unclear governance structures (nationally and at-facility-levels), lack of representation public health experts in management teams, weak delegation of authority at hospital-level, and limited engagement at community-level. In the first months, KIs reported that many EMR hospitals were refusing to deal with COVID-19 and pushed for a centralized response, in the capital cities and in designated COVID-19 facilities. In cases where decentralization was used without adequate coordination of stakeholder, the duplication of plans and the lack of clear guidelines contributed to fragmentation and disorganization in the response. Coordination with the private sector was a reported challenge in many countries. KIs reported that some private hospitals, especially in the Region’s LMICs, were initially willing to give their beds and ventilators, train staff, and give money instead of receiving suspected patients. Moreso, in some cases, the disengagement and limited regulation in the private sector resulted in poor implementation of clinical guidelines and skyrocketing prices for testing and treatment for COVID-19.

To optimize their response, multisectoral collaborations at national and facility-level were utilized across the Region bringing together the private sector, educational institutions, military, national and international NGOs. The role of hospitals and representation of HMs and senior clinical experts in national response committees was essential to policy development, planning, and creating national guidelines for the response [14]. Hospitals across the Region further highlighted the importance of strong hospital networks, integrated within a PHC-system, and emphasized the engagement of community actors and local partners in the COVID response coordination. This study revealed 82% of respondents confirmed the availability of a coordination mechanism between the hospital and other local partners, actors, and other organizations, indicating the importance of strong hospital networks and engagement of community actors in the COVID response. These networks enabled prompt action in implementing interventions, increased community ownership, and empowered hospitals across the EMR to respond more independently and efficiently [44, 48–52]. In many countries, HMs of larger tertiary teaching hospitals noted their role in building the capacities of their peripheral counterparts through virtual trainings, online-consultations, 24/7 hotlines, in-service trainings, dispatching staff and lifesaving supplies to support critical cases, and sharing, and cross-checking protocols adapted from accredited hospitals.

More than half of countries in the Region, including Afghanistan, Iran, Iraq, Jordan, Lebanon, Morocco, Oman, Pakistan, Saudi Arabia, Somalia, Sudan, and Tunisia utilized Public-Private-Partnerships (PPPs) in the COVID-19 response. Hospitals in the EMR utilized PPPs to expand service delivery, strengthen referrals, increase lab capacity, improve staffing, financing, procurement, and operations, as well as develop and update clinical guidelines [53–55]. Hospitals also played a great role in educating and communicating with the public, and coordinating other community stakeholders [7, 56].

Furthermore, the role of HMs and strong leadership was among the most cited themes and confirmed as a key enabler to hospital response, readiness, and resilience [29, 47, 57]. Qualitative data defined this strong hospital leadership as ‘participatory’, ‘reliant on teamwork’, ‘trusting in technical expertise of others’, ‘motivating staff’, ‘adaptable’, ‘supportive’, ‘sacrificial’, ‘decisive’, ‘respectful’, ‘clearly communicating vision’ and ‘strategic’ particularly in times of crisis.

### Supply management, operations and finance

**Fig 3:**
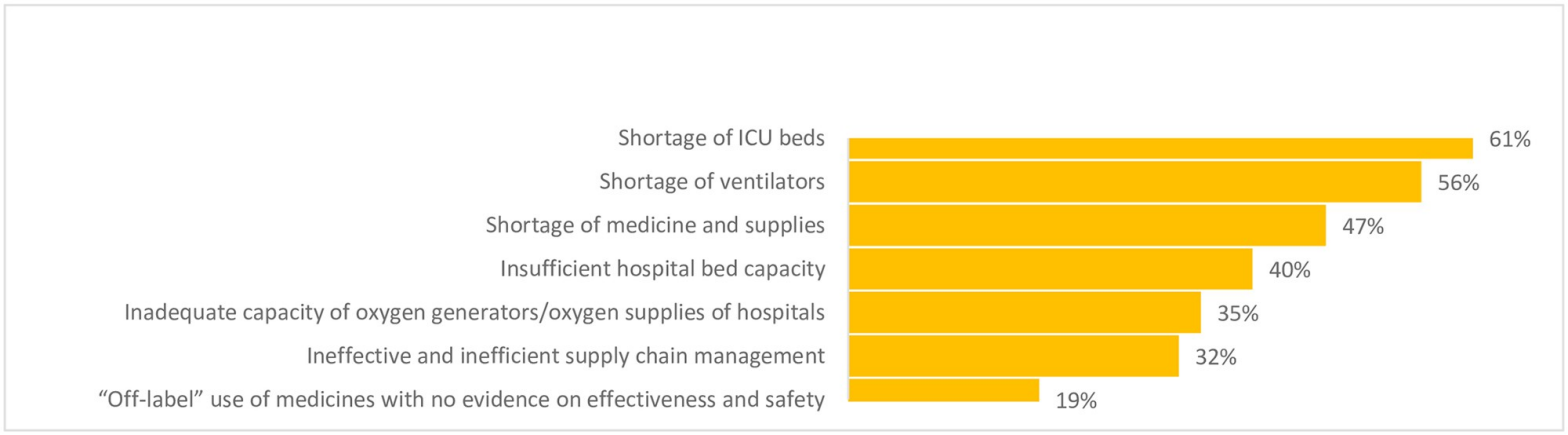
Challenges of hospitals in the EMR related to medicines, supplies, equipment (n = 139), July 2020.

In many of the Region’s LMICs, the closure of businesses, travel, and trade, resulted in the exacerbation of financial crises [11]. In the first months of the pandemic, many hospitals faced increased costs as they prepared for surges of COVID-19 cases while concurrently suffering from revenue losses due to the discontinuation of elective procedures and other services. The shortages of PPEs in the first months of the response were unilaterally reported by hospitals in high-income, LMICs, and FCS while the shortage of oxygen supply was the most frequently cited theme in FCS. Our survey revealed that hospitals’ top reported challenges to operations included the lack of financial resources (59%), shortages of PPEs (58%), weak IPC (56%), and hospitals overcrowding (53%). The shortages of beds, ventilators, and medicines were frequently reported (Fig 3) while the shortage of oxygen supplies was exacerbated in FCS and cited by KIs in Yemen, Somalia, Sudan, Libya, Iraq, and Afghanistan.

These scarcities and disruptions to procurement were often exacerbated by the lack of a systematic approach to monitor or assess inventory, stockouts and orders both at facility and national levels, weak governance and information structures, unregulated markets, and centralized procurement. In FCS, KIs noted the disrupted supply-chain mechanism, insecurity, donor-dependency, and fragmentation resulted in delayed procurement. In some LMICs and FCS, KIs highlighted the lack of transparency in resource allocation also contributed to competition between ministries, hospitals, and other actors, and increased distrust in hospital management and government. KIs also reported that some hospitals competed for supplies due to maldistributions, poor quality of PPEs despite high prices, and overuse and misuse of these limited resources, and delays in confirming suspected cases. KIs from LMICs noted the inequitable allocation of resources because of delays in planning and fragmentation resulted in disruptions to services provision, poor staff retention, and overall distrust in leadership.

To overcome these challenges, HMs utilized innovative solutions to diversity their financing mechanisms citing the need for flexible and autonomous funding to facilitate readiness and response. Some designated hospitals in the EMR reported receiving additional funding from ministries of health (MOH), in other cases this support was not a cash-flow injection but rather cutting additional costs of procurement or covering referrals. Across the Region, few hospitals implemented free treatments for COVID-19 and reduced fees for those on public insurance or welfare; this intervention requires further considerations needed to cover migrants, refugees, and displaced populations. In other cases, hospitals utilized PPPs or obtained additional financial support from the private sector, corporate and community donations, military, and NGOs, and pooled their budgets and procurement efforts.

Regarding supplies and logistics, KIs applied early procurement ahead of global inflations in PPE prices, allowed smoother operations and improved IPC, especially in the early months. Some hospitals utilized needs-based assessments, inventory checks, and in-house storage for surges, and risk-based distribution of resources to manage the limited availability of supplies and estimate needs. Our survey found this intervention was reported by 50% of participants in 14/22 countries.

***“But we have what’s called buffer stock in our hospital in case of any disaster*. *We were assessing this stock in advance*, *and this really helped us later as we faced the initial shortages*, *and the shortages due to over-utilization of PPEs at the beginning*, *and now*, *especially as wearing surgical mask and others is supposed to be mandatory for all healthcare workers inside the hospital and even in public.”***

***(KI-16)***

To reduce reliance on disrupted and expensive international supply chains, many EMR hospitals also relied on self-production or purchasing locally made supplies.

### Communication and information

At the beginning of the pandemic, HMs in many EMR countries described the widespread rumors, stigma, and misinformation as chief challenges to the response. KIs further reflected on the impacts of the media in the early months (March-July 2020) on spreading false health information to both patients and health workers, reinforcing negative messages about the safety of hospitals facilities, and altering the public’s perception, distrust, utilization of health services, and adherence to IPC measures. In the early months, HMs warned against deviations from evidence-based clinical management as many health workers sought their information and guidelines from media. One KI noted: ***“A lot of doctors believe and apply rumors from WhatsApp and Facebook to treat patients*. *There was also a rumor that isolation centers are using the mercy killing of COVID patients; patients were refusing to be admitted” (KI-43)*.** Nowadays, public distrust of hospitals and health systems continues to be a challenge in the COVID response. Moreover, public misinformation related to risks, symptoms, and severity increased hospitals workloads during critical times while the stigmatization of hospitals due to the high infection rates, overcrowding and under-equipment resulted in delayed health seeking behavior until patients were severely critical. In some cases, poor inter- and intra- hospitals communication also resulted in fragmentation and inefficiencies in the response.

In response, EMR hospitals have learned the significant role of community during outbreaks [22, 58, 59], one KI emphasized: ***“To win this pandemic*, *to win this battle*, *we have to win the community*, *the community is the key*.*” (KI-14)*.** HMs utilized a variety of interventions to combat stigma, regain public trust, and raise awareness including assigning official spokespersons, designating a media and communications team, mobilizing volunteers, broadcasting daily news briefings, publishing multi-lingual flyers and educational posters, using hotlines, webpages, and social media accounts, creating applications for screening and health education, and engaging religious and community leaders [44, 49, 60–63].

KIs also noted that improving vertical and horizontal communication between health workers and hospital management, and especially among various specialties, teams, and departments, resulted in improving teamwork, morale, efficiency, and health worker knowledge of preparedness plans and updated clinical and IPC protocols. In our study, 42% of respondents reported daily updates while almost 30% reported receiving COVID-19 information weekly. Across most EMR hospitals, the use of technology, virtual and remote communication was reported whether through Whatsapp groups for staff and diaspora health workers, zoom meetings, or hospital electronic management systems.

***“Now Whatsapp is widely used for a doctor to help a doctor having to deal with difficult situations*. *Whatever new updates that come out*, *there’s regular zoom meetings with WHO*. *There was also CMEs or continuing medical education that was shared [online] by the Medical Association about guidelines*, *follow-up*, *or updates together with the standard operating procedures.”***

***(KI-29)***.

Finally, inter-hospitals communication and regular meetings with local and national authorities were reported to receive feedback and assessments from MOH, learn from experiences of other hospitals, optimize beds availability, and improve coordination, referral capacity, and critical care expertise.

### Human resources

**Fig 4:**
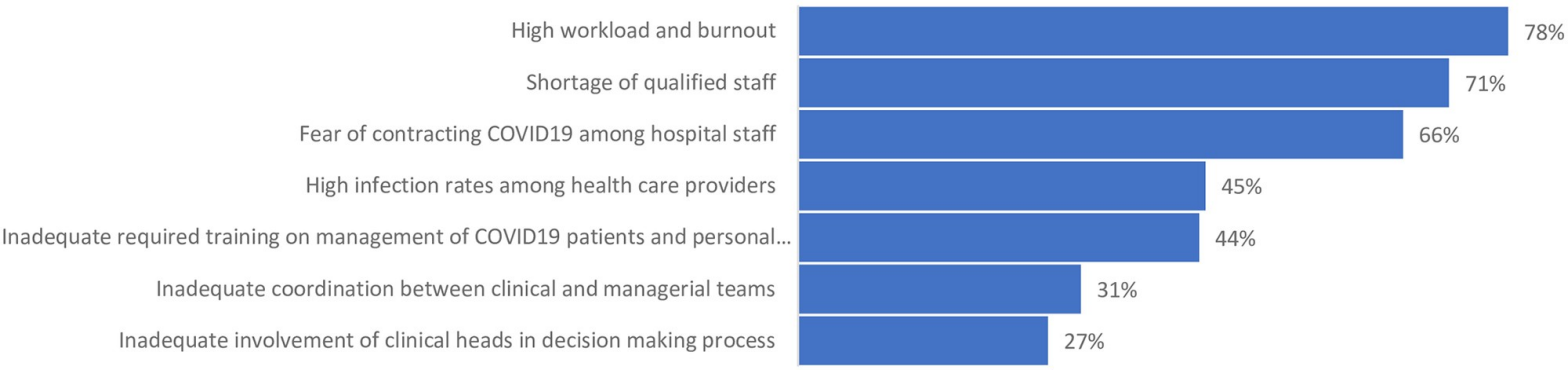
Challenges faced by hospitals in the EMR related to human resources (n = 139), July 2020.

Managing hospitals workforce during this pandemic essential to health systems’ response and resilience [64, 65]. Across the Region, prior to the pandemic, about a third of countries suffered from critical shortages of health workers while more than half of countries, especially in LMICs and FCS, faced labor market maldistributions, whether in skill mix, gender, geography, sector, or level of care [66]. In responding to the pandemic, hospitals in the Region’s LMICs, reported that one of the biggest HR challenges was the shortage of critical care, infectious diseases, emergency, radiology, IPC, and respiratory specialists and nurses. These shortages of specialists were critical in the Region’s FCS, confirmed by KIs, survey and literature findings [36, 46, 67]. In a few countries, due to the shortages of nurses, hospitals relied on patients’ relatives to provide support services which contributed to cross-infections. Additionally, these shortages added to an increased workload and pushed HMs to increase staffing for surge through various recruitment measures, although some were unsuccessful due to the harsh financial crises. KIs in LMICs reported various challenges to HR management contributing to weak retention of critical staff; in some of these countries, these shortages were exacerbated by some specialists refused to work in COVID-19 centers whereas in others, some private hospitals fired staff exposed to COVID-19. KIs further reported that inconsistent remuneration, insufficient incentives, delays in payments for public-sector HR, limited job security, high workload, risks of exposure, infection and mortality, shortages of PPEs, distrust in IPC measures (nationally and at facility level), violence against front-liners, and limited training contributed to health worker demotivation, burnout, absenteeism, and poor retention. In our survey, 78% of respondents reported high workload and burnout as the main HR challenge in hospitals, 70% reported the shortage of qualified staff, followed by fears of contracting the virus and high infection rates (Fig 4). Across the EMR, findings showed female health workers and more junior staff in training were more affected by stress, burnout, and anxiety while limited mental health and psychological support was provided for nursing and paramedical staff compared to physicians [35, 68, 69].

In addition to this, findings showed health workforce attitudes regarding the virus, the lack of adherence to evidence-based guidelines, poor communication, and over-reliance on inexperienced staff to respond to surges weakened hospitals responses [70, 71]. In several LMICs, the shortages of hospital workers required recruiting new and inexperienced staff to support the response, these health workers were often learning on the job. One policymaker reflected: ***“Some did not have competencies or experience*. *I interviewed them and some did not work even a single day in the hospital; how these [junior] people manage such a big crisis of COVID-19*?*” (KI-1)*.** Moreover, this study’s qualitative findings further revealed that insufficient training described as ‘too short’, ‘too generic’, or ‘not tailored to critical care or specific cadres’ which affected the poor clinical competencies of front-liners. On the other hand, HMs confirmed the need for continuous sensitization and training of hospital workers on IPC, use of PPE, critical care, and emergency management.

In response, hospitals across the EMR implemented numerous interventions ranging from recruiting surge staff, providing incentives, protecting staff health, and building capacities. Firstly, across the Region, hospitals utilized various interventions to increase available and qualified staff, our survey indicated that 80% of hospitals in 14/22 countries redistributed staff according to needs and surges. HMs reported reassigning staff from other departments/specialties or from PHC, task-shifting (and training) to different specialties, recruiting low-risk (often junior) staff for ICU, hiring international staff for surge, recruiting volunteers, retirees, and fresh medical graduates [44, 48–52]. HMs also reported deploying emergency medicine, critical care, respiratory, cardiology, and internal medicine specialists to ICUs to support COVID-19 response.

Secondly, regarding incentives, the highest reported in our survey was recognition and acknowledgment (63%), followed by financial (53%) and non-financial incentives (38%). Most KIs reported hospitals providing meals, accommodations, and transportation for front-liners in designated hospitals. In LMICs, HMs reported extending annual leaves, paying over-times, increasing staff salaries, while in few FCS, hardship payments and additional incentives were given. In the early months, HMs from LMICs proposed financial incentives however reflected on their ineffectiveness in retaining and motivating front-liners especially given the reductions in hospitals’ revenues, high workloads, and risks.

Thirdly, in protecting staff health and motivation, most designated hospitals implemented a two-week rotation in staff to reduce risk of cross-infections; however, some hospitals in LMICs faced difficulties due to the shortages of health workers. According to our survey, the top interventions used by EMR hospitals in protecting and maintain staff health included daily distribution of and training on PPE use (94%), regular risk assessments and screenings (63%), and dedication of specific accommodation and transportation for staff with high exposure (62%). Some EMR hospitals provided staff and their families free testing and treatment if infected. In some specialized hospitals managing high-risk patients, health workers were restricted by zones to avoid cross infections, as reported by a KI referencing a specialized cancer hospital: ***“Staff working with the elderly*, *patients with chronic diseases and cancer were not allowed to enter other departments*, *especially those providing chemotherapy and kidney dialysis*, *to reduce risks for both patients and health workers” (KI-18)*.** Furthermore, KIs across high and LMICs noted the importance of promoting mental health and psychosocial support, whether disseminating tips and guidelines on self-care and mental health through informal WhatsApp groups, or designating psychologists for COVID-19 front-liners. In few countries, hospitals recruited young and early career psychiatrists to support health workers [35, 38–40, 72]. Nevertheless, these interventions require further investment and emphasis, particularly in preparing for subsequent waves and prolonged response [73].

Finally, in preparing health workers to respond to COVID-19, hospitals in the EMR needed to provide a comprehensive training to protect health workers from the virus and raise their competencies in providing critical care, especially with the continuously changing evidence on the nature of the virus, its epidemiology, infectivity, transmission, and treatment. Our survey revealed that across 2/3^rd^ of countries in the Region, the most reported trainings were on proper use of PPE (92%), followed by trainings on case definitions and transmission (88%), IPC (86%), screening and triage (86%). Hospitals across the Region utilized technology, virtual trainings, and social media platforms to train and re-train multi-disciplinary teams, including custodial and non-medical hospital staff, as highlighted:

***“COVID does not respect artificial specialties*. *The orthopedics had to start learning how to deal with COVID patients*, *the plastic surgeons had to learn how to deal with COVID patients*, *the medical orderlies*, *the cleaners in the hospital had to be educated and to be taught how to learn with this new disease; this involved a lot of training and retraining.”***

***(KI-15)***.

While many hospitals across the EMR utilized online and virtual trainings, as well as short videos and educational materials shared on social media such as Whatsapp Groups or Medical Association Facebook Groups, continuous capacity building on leadership, communications, critical care management and IPC were highlighted needs by many KIs.

### Surge capacity and essential services

Across the Region, hospitals attributed disruptions to essential health services (EHS) to the stigmatization of the health system, fear, distrust, and underutilization by the public, decrease in outpatient volume, closure of health facilities cancelations in elective procedures and disruptions in referral pathways. These disruptions complicated care for critical and chronic patients while expensive and inaccessible testing and overcrowding of hospitals in many countries resulted in confusion and delays in seeking care for non-COVID patients: ***“There is a high mortality rate in non-COVID patients*, *they don’t have good care because hospitals can’t receive patients because elective and outpatient clinics are closed*.*” (KI-8)*** In the EMR, the types of services that were least disrupted were emergency and critical care, especially in FCS as trauma cases continue to require care despite surges in COVID-19 cases [74]. In these countries, the provision of essential health services was further challenged by insecurity, lockdowns, and limited mobility of both patients and health workers [44, 48, 75].

Maintaining EHS was critically important in hospitals’ responses to COVID-19, most HM reported creating guidelines to maintain critical hospital operations and services through emergency departments for at-risk patients, particularly those needing continuity of care (e.g. parturient, cancer and hemodialysis patients) [76, 77]. A KI from a specialized cancer hospital shared: ***“Every cycle chemotherapy session is necessary for their survival*. *You can’t stop it one day*, *then return back after three days*. *All these things were kept in mind when trying not to disrupt essential services*.*” (KI-19)*.**

**Fig 5:**
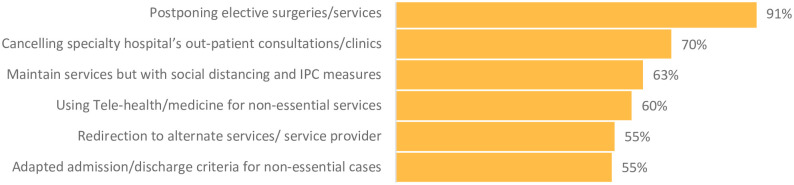
Strategies to manage non-essential patient flow in hospitals in the EMR (n = 139), July 2020.

According to the survey, about two-thirds of respondents indicated that hospitals established strategies to maintain services for at-risk patients while 80% reported hospitals suspending non-essential services among other interventions to manage patient flow and increase capacity for surges (Fig 5). The establishment of field hospitals and use alternate or secondary care sites, such as gymnasiums, hotels, community centers, were other measures implemented in most countries [78].

The use of telemedicine especially in maintaining non-COVID health services was reported by all KIs, highlighting the importance of this intervention in hospital response to COVID-19.

***“We treat a lot of cases*, *more than thousands at home*, *on oxygen*, *on steroids*, *and follow-up by mobile*, *WhatsApp*, *Viber*, *Messenger*, *24 hours live; we are busy but the people are in a bad situation*, *we have to help them.”***

***(KI-8)***.

In response to surges, many hospitals in the EMR increased the availability of ICU beds by purchasing and building ICU with new equipment and beds, converting emergency departments into isolation wards and treatment centers (e.g. most countries in EMR), early discharging of emergency patients to clear out ICU beds (e.g. many LMICs) and collaborating with private sector to increase available beds and space for treatment [50, 52, 79]. Additionally, prior to the adaption of global guidelines, several hospitals in LMICs utilized home visits to treat mild and moderate COVID-19 cases which reduced hospital loads, expanded available bed capacity for severe cases, maintained patient flow, and provided EHS. This intervention was widely applied as early as April and May 2020 in some of LMICs (especially in FCS), due to the limited bed capacity and the need to ration resources appropriately.

### Identification and diagnosis

In the early months of the response, hospitals across all EMR countries reported severe shortages of testing supplies and extended waits for test results, due to limited and centralized testing capacities, which caused unnecessary hospital admissions and overcrowding in designated hospitals [50, 52, 79]. Additionally, these inefficiencies in diagnosis resulted in longer lengths of stay in hospitals (especially for suspected cases waiting admission or treatment), delays in discharging patients, extra use of limited PPEs and inability to utilize bed capacity efficiently [75, 80]. Moreso, the shortages of qualified lab personnel in many EMR countries exacerbated detection and diagnosis [81].

According to our survey, respondents revealed that 91% of hospitals had a functional x-ray machine, 75% had CT-scans, 53% had MRIs and 60% had hospital-based PCR testing capacities. KIs further reported distrust in the reliability of PCRs resulted in clinicians in many LMICs relying on other diagnostic tools, such as CT scans or chest X-rays, or other clinical signs and symptoms to make a diagnosis: ***“As a clinician*, *I trust high resolution CT scans more than PCR in severe cases because of the testing limitations” (KI-34)*.** This weak and centralized diagnostic capacity along with the shortages of qualified lab personnel especially in FCS, resulted in huge under-estimation of cases and under-reporting of incidence and mortality; this false epidemiological assessment further inhibits adequate planning, procurement of resources, deployment of staff, and provision of necessary and critical care [81].

Regarding information, numerous LMICs, especially in FCS, hospitals face challenges in reporting standardized, timely, comprehensive, accurate data, due to limitations of national health information systems, political differences, and lack of coordination in requesting different statistics from relevant authorities at different levels created fragmentation and inconsistencies in reporting.

In response, across almost all countries in the EMR, scaling up and decentralizing national testing capacity was among the chief interventions [81]. Hospitals in the EMR increased their capacity for rapid identification and diagnosis through establishing fever clinics outside the main entrances of hospitals, setting up separate triage and screening areas for suspected cases, and establishing in-house laboratories [40, 76, 82]. The establishment of hospital-level PCR labs was reported by KIs in Syria, Saudi Arabia, Pakistan, Palestine, Oman, Iran, Kuwait, and Bahrain. This intervention was easier for accredited hospitals, which had existing infrastructures and strategies to improve quality, safety, efficiency, and patient-centeredness. The proactive institution of facility-based PCR testing improved rapid identification and diagnosis. KIs shared: ***“Our hospital also was the first to introduce the PCR test*. *In the month of February*, *we introduced the gold standard PCR as we are CAP accredited*.*” (KI-26)*** and ***“We built our own micro-laboratory to make PCR available at our hospital*, *so we could confirm our diagnoses*, *define cases*, *and manage the outbreak in our facility” (KI-19)*.** These facility-based PCR labs not only improved and decentralized testing capability, but it also helped improve with case management, reduce delays in triage and treatment, ameliorate early diagnosis and referral, provide free hospital beds, improve patient flow, reduce overcrowding in designated hospitals and improve overall efficiency.

To improve rapid identification and diagnosis, hospitals issued protocols and updated clinical guidelines in line with the latest evidence. Our study revealed, the most commonly available protocols in EMR hospitals were for screening and triage of suspected cases (94%), followed by reporting suspecting and confirmed cases and deaths (86%). Additionally, almost all countries in the EMR applied contact tracing while some used digital platforms to monitor epidemiological surveillance, detection, reporting, and notification at national and/or facility-levels. Hospitals further optimized capacity for diagnosis by partnering with private laboratories, expanding tele-screenings, using mobile and drive-through testing, allocating flexible spending towards procurement of test kits for hospitals, or training additional lab personnel.

### Isolation and clinical management

Overwhelmed designated hospitals was the most frequently reported challenge to hospitals operations and clinical management.

On the one hand, KIs listed numerous health systems and supply-side factors contributed to these strains including but not limited to: under-preparedness of hospitals to respond to surges, lack of flexibility in changing hospital infrastructure, limited number of ICU beds, delays, costs, and inefficiencies in testing, rapid rises in surges and the changing nature of the virus and its infectivity. These shortages of COVID-19 hospitals, ICU beds and ventilators were frequently exacerbated in FCS; additionally, hospitals in LMICs reported limited quarantine or isolation spaces for suspected cases in the hospitals [11, 52, 75, 83]. Hospitals were further challenged by weak referral pathways and severe shortages of essential resources (specialists, medicines, and oxygen) exacerbated in FCS [36, 65]. On the other hand, KI reported several demand-side challenges threated clinical management further straining hospitals operations such as patients’ resistance to quarantine due to stigma and costs, delays to seeking care until critical, and frequent changes between hospitals due to high costs in the private sector (often reported in LMICs).

Among the chief threats to COVID-19 response was the lack of adherence to evidence-based medicine across EMR hospitals. In some LMICs, KIs attributed inconsistent, conflicting, or lack of guidelines established at national level as the main contributing factor. Others suggested the pressure from patient relatives and general community resistance to IPC measures and isolation due to culture, stigma, and misinformation. The lack of health worker knowledge of constantly changing protocols and their reliance on myths from social media, resulted in some clinicians implementing their own variations of treatment measures, including the use of off-label medications [41, 70, 71, 84, 85]. A HM confirmed the regional literature, recounted:

***“There was no proper case management guidelines [at the hospital]*. *There was an argument between the doctors*, *specialists*, *everyone was deciding on his own interventions*, *depending on information from the media*, *from Whatsapp*, *from Facebook to manage COVID cases”***

***(KI-30)***.

To increase capacity for isolation and critical care management, hospitals across the EMR allocated separate buildings outside the main hospital to quarantine suspected cases and collaborated with other sectors to convert hotels or unused clinics, schools, malls, and facilities. This intervention was applied widely as mentioned by KIs from Afghanistan, Bahrain, Kuwait, Libya, Palestine, Pakistan, Saudi Arabia, Somalia, Sudan, Syria, UAE, and Yemen, confirming the literature findings. HMs in most countries further resolved to applying zoning, using field hospitals, purchasing new equipment and ICU beds, or in many LMICs, discharging ICU patients early to increase capacities. In the later months of the response, hospitals across the Region only admitted critical and severe patients and created stricter triaging protocols to reduce hospital load, improve operations, and ration limited supplies [86–88].

To improve clinical management, many EMR hospitals mandated in-service training on critical care management and utilized virtual and on-the-job trainings. In 8/22 countries, KIs highlighted innovative capacity building methods utilizing telelearning to provide in-service training on critical care management and setting up ICU teleconsultations hotlines to support district hospitals: ***“Our hospital created an online consultation service (hotline) for the government intensive care doctors; they call anytime and there is immediately information-exchange and technical support*.*” (KI-26)***

Additionally, hospitals in the EMR used other innovative clinical approaches, such as opting against intubation and using less-invasive therapies, as reported by HMs from Bahrain and Iraq as early as June and July 2020. HMs reported adapting clinical guidelines based on facility-level, national, and global experiences: ***“We adapted and pushed more for high frequency*, *noninvasive ventilation and high flow oxygen therapy*, *which is different from Italian recommendation*.*” (KI-3)*** In some FCS, innovative practices were needed to address the severe shortages of oxygen supplies in ICUs; a notable example is seen in Somalia’s use of solar power to increase oxygen in hospitals [89, 90].

Moreso, across the Region, the development of national and facility-based scientific committees guided the creation, revision, updating, and dissemination of the latest clinical guidelines based on international standards and in line with new, growing, and evolving evidence [73]. KIs from 10/22 EMR countries reported assigning a team to review the latest literature and update clinical protocols at facility-level. In over a third of EMR countries, KIs noted MOH closely monitored the compliance and implementation of these guidelines in facilities.

### Infection prevention and control

At the beginning of the response, the newness of the virus, poor compliance, high infection rates, and overuse of PPEs exacerbating global and regional shortages were among the biggest threats to IPC. Other factors included weak referral pathways of suspected cases, mismanagement of visitors, inadequate health worker knowledge and training on PPE use, and weak and nonagile infrastructure to secure staff and patients’ safety.

In many LMICs, especially those in FCS, KIs noted that the lack of IPC policies nationally and at-facility level hindered patient and staff safety in hospitals, especially in the early months where PPE shortages were common. In some of these countries, KIs explained there may be national written or documented plans and guidelines related to IPC however, the implementation, adherence, and evaluation at facility and national levels were weak. Moreso, KIs confirmed the literature findings and further noted the culture of negligence around IPC in hospitals where despite an understanding of guidelines, provider behaviors show misuse and lack of adherence [49, 64, 67, 91, 92]. In 12/22 countries, KIs revealed the biggest challenges to IPC in hospitals was provider knowledge of guidelines, their limited ability to use PPE, and their poor adherence to protocols.

In the early response, the mismanagement of visitors was another major cause for spread of infections in hospitals. In several LMICs, the shortages of nurses resulted in some hospitals allowing companions to support the provision of non-clinical support to patients. Others reported the weak implementation of this IPC measure was due to cultural reasons:

***“Culturally*, *it is not acceptable [to stay away and not care for the sick]*. *Because of the small number of healthcare workers working in the ward*, *patients are obliged to bring somebody to accompany them*. *So the [patient’s relatives] were dealing with this patient*, *doing the nursing jobs*, *cleaning them and doing the basic necessary things*, *feeding them*, *giving them the baths.”***

***(KI-30)***

**Fig 6:**
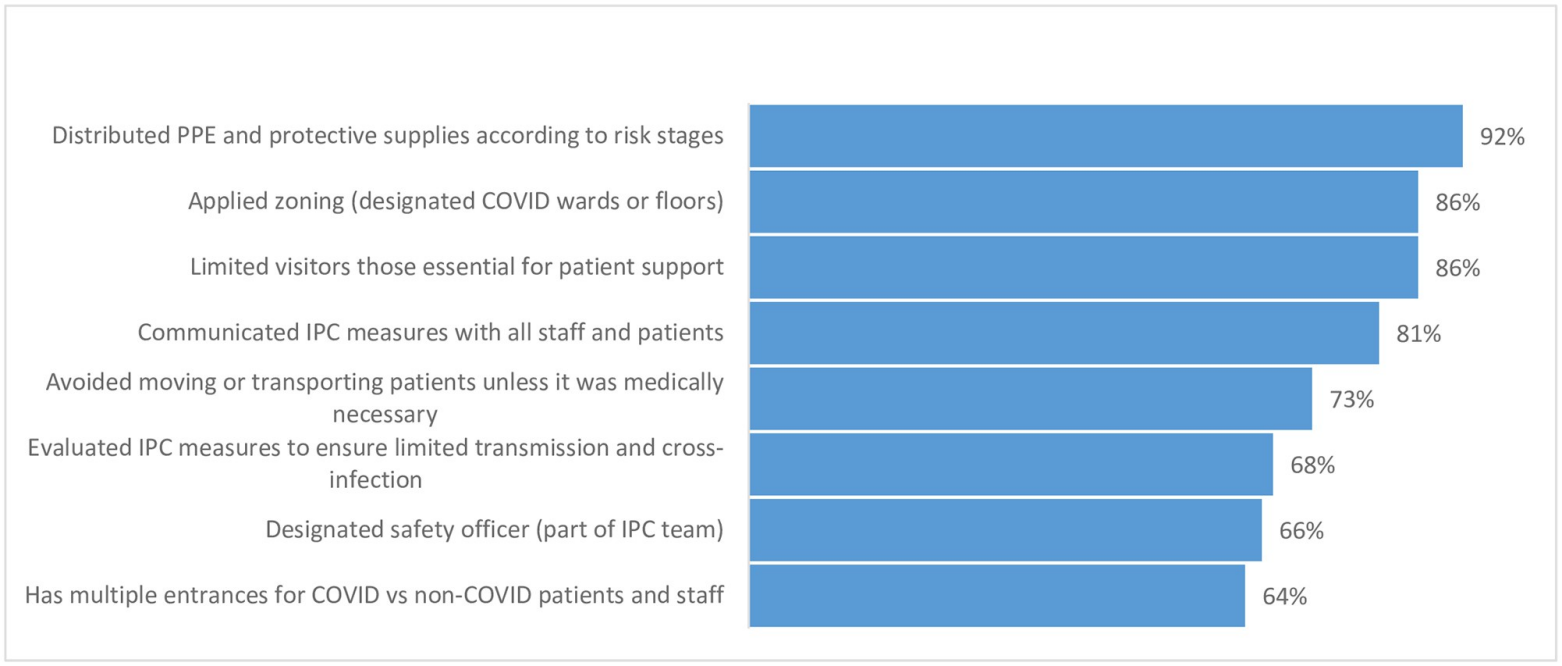
Interventions to limit the nosocomial transmission of COVID-19 in EMR hospitals (N = 139).

In improving IPC and limiting the spread of nosocomial infections, hospitals in the EMR implemented numerous and frequently changing interventions (Fig 6). In more than half of EMR countries, hospitals reported daily distributed PPE and protective supplies according to risk.

KIs further highlighted the importance of hospitals’ agility in changing hospital infrastructure to create spaces for IPC measures (i.e. donning, doffing, screening, triaging suspected cases) and designating specific COVID-19 wards or floors for their treatment. HMs confirmed literature findings with examples from HMs in Jordan, Kuwait, Iraq, Oman, Palestine, Pakistan and Saudi Arabia [44, 49, 60, 93, 94]. Establishing and communicating IPC guidelines, limiting visitors, and designating IPC officers in hospitals were among the most reported interventions across the Region. In hospitals where safety officers were not available or assigned, the role of nurses was central to implementing and evaluating IPC measures:

***“The availability of qualified infection prevention practitioners*, *especially nurses were very helpful*, *because they were in charge of quality and infection control*, *along with accreditation which allowed some facilities to be ready for this pandemic.”***

***(KI-9).***

Nurses and IPC officers across the Region were responsible to improve quality and safety, reduce infections, train other health workers on proper use of PPEs, and monitor the compliance with IPC measures [95, 96]. Moreover, our survey found that more than 50% of participants agreed that their hospitals implemented strict supervision regarding the implementation of IPC measures. Many KIs reflected on the improvements to IPC from the earlier months to the later ones due to increased training and availability of PPEs.

Furthermore, in hospitals with quality assurance mechanisms, implementing accreditation or PSFHI initiatives, KIs highlighted increased preparedness, response, efficiency, safety, and IPC. HMs further concluded that accredited hospitals in the Region often had better preparedness, smoother hospital operations, and stronger IPC. In the face of subsequent waves, systematic efforts to improve IPC and investment in EMR hospitals preparedness and resilience are necessary to ensure staff and patients safety while maintaining essential functions.

## Discussion

This study was conducted to explore hospitals experiences in combatting COVID-19 across the EMR with the purpose of providing context-specific evidence and recommendations for policymakers and HMs. This thematic analysis revealed numerous challenges and interventions for each of major hospitals readiness domains, namely: preparedness, leadership and coordination, supply chain management and operations, communications and information, human resources, surge capacity and essential services, rapid identification and diagnosis, isolation, and clinical management, and finally, infection prevention and control (Table 1).

**Table 1:** Summary of main themes for EMR hospitals’ challenges and interventions by checklist domain.

Domains/Themes	Challenges	Interventions
**Preparedness**	Newness/stigmatization of virus No holistic or system-approach Poor preparedness for surges Inefficient allocation of resources Parallel governments: inconsistent/ opposing guidelines and weak implementation Fragmentation & duplication of interventions [FCS]	Anticipate surges = proactive action Use multi-sectoral/specialty EOCs Build on measures from past outbreaks (MERS, SARS, …) Agility, adaptability, and flexibility in implementation (i.e., interventions changing over months to respond to evolving challenges)
**National/Sub-National Levels**	Delays & inefficiencies in planning Weak emergency response structures Poor implementation of national plans	National preparedness and response plans Assessments of health system and hospital sector readiness
**Hospital Level**	Initial resistance to deal with COVID-19 Limited plans at hospital levels (i.e., Emergency plans not tailored for outbreaks or plans on paper but not in practice)	Hospital-wide codes, simulation exercises, daily risk and epidemiological briefings Piloting interventions in one designated hospital Role of hospitals accreditation/PSFHI in preparedness and response
**Leadership**	Politicization of Response Poor coordination of stakeholders: Duplicated efforts & conflicting policies (FCS) Disrupted procurement, inconsistent protocols, inequitable resource distribution Decentralization without coordination Private sector unregulated/disengaged Lack of awareness of situation on ground Lack of engagement of public health experts Limited autonomy of hospital managers on resource management and operations Weak leadership capacities of hospital managers	Multisectoral and multispecialty response committees Representation/Role of hospitals EOCs at hospital-level Public-Private Partnerships (i.e., Expanding service delivery/referral, Staffing and financing, Sourcing supplies, beds, facilities, Capacity building) Capacity building of peripheral hospitals (i.e., Virtual consultations/trainings, Dispatch staff and life-saving supplies to support critical cases, Cross checking protocols) Role of hospital managers (i.e. Participatory and adaptable, Delegation, Accountability)
**Operational support, logistics, supply management**	Shortages of supplies/ PPEs, ICU beds, ventilators Shortage of oxygen supply in FCS No systematic approach to monitor inventory/needs/stock-outs Increased costs but limited revenues Disrupted SCM: Fragmentation and Delayed Procurement	Centralized procurement & distribution Needs-based and Inventory-checks Early procurement Obtaining supplies locally Local and self-production & Donations Diversifying funding and hospital financing mechanisms: Different funding sources: Dedicated MOH (55% of surveyed), Military, NGOs (FCS), Private donations More flexible and autonomous funding at hospital-level Free treatment in public hospitals, adjusted costs for welfare, insurance schemes
**Communications and Information**	Public distrust Media spreading rumors, stigma, misinformation Poor health information system	Communication with the public (i.e. Hotlines, Call centers, prints, messages, social media, Engaging religious and community leaders, Daily news reports, Designating official spokespersons for hospitals, Using mobile applications and GIS) Communication between health workers (e.g. WhatsApp, note Role of diaspora (LMICs and FCS)) Communication between staff & mgt Communication between hospitals & MOH
**Human Resources**	Shortage of critical care, infectious diseases, emergency, radiology, IPC, and respiratory specialists and nurses (especially in LMICs/FCS) Increased workload Weak planning for renumeration and incentives, Inconsistent payments, Poor recruitment/ retention mechanisms HRH fear and perception of virus, causing: Absenteeism, Anxiety and burnout, High infection rates (PPE shortages), Violence vs. front-liners (FCS) Weak qualifications and Inadequate training on IPC, critical care, Management Poor competencies of New and inexperienced staff	Recruit for surge (i.e., Better HRH planning and distribution based on need/surge, Task-shifting/training different specialties, Hiring international staff, Volunteers, retirees, and fresh medical graduates) Protecting and maintaining staff health (PPEs, 2-wk Rotations, Insurance coverage, free testing, Psychological/emotional support, Designated psychologists) Incentives: Recognition, Financial, Non-Financial (e.g. meals, accommodations, childcare, transportation) Training in PPE, Screening/Triage, ICU/Clinical Mgt, Multidisciplinary Trainings Using tele-learning and social media
**Continuity of Essential Services and Surge Capacity**	Closure of health facilities Stigmatization of hospitals Disrupted referral pathways and EHS Increased complications, outbreaks, deaths Confusion for non-COVID pts	Strategies and Guidelines to maintain EHS (WHO guide) Reducing hospital loads and managing patient flow (i.e., Postpone electives and close OPDs, Maintaining only emergency services, Relying on PHC/referral pathways) Establishment of field hospitals and use of alternate care sites Telemedicine for management of EHS and non-COVID cases and home-treatment of mild/moderate COVID Established public dashboard for COVID 19 and using electronic surveillance and information systems
**Rapid Identification and Diagnosis**	Limited and centralized testing capacity affected by shortage of kits, shortage of qualified lab personnel, inadequate triage and isolation spaces for suspected cases Delays in PCR Results resulting in limited bed capacity, longer LOS & waiting times, shortage of PPE, and reliance on CT, XRays, clinical signs/ symptoms	Scaling up testing capacity Establishing in-house PCR labs Decentralizing testing Mobile clinics, community swabs Rapid procurement of testing kits Flexible funds at hospital-level Tele-screening Fever-clinics at points of entry and hospital entrances
**Isolation and Case Management**	Designated hospitals overwhelmed (limited spaces for isolation and treatment) Rapid rise in cases/changing nature of disease Weak referral systems Evidence-based medicine not adhered Patients’ delays to seeking care until critical High and unregulated costs in private sector Shortages of specialists, medicines and oxygen	Designating COVID-19 facilities (i.e., Space outside main hospital buildings, zoning, Converting wards for ICU/triage/isolation, alternate care sites, PPPs, Purchasing new equipment/beds for critical care) Increasing ICU technical capacity (i.e. Updating and disseminating guidelines, Training and hotlines for ICU staff, Early discharge to clear ICU beds) Innovating clinical approaches: Increasing oxygen supply [FCS] Using digital health (teleconsultations) Opting against intubation/ using less invasive therapies Establishing quality assurance mechanisms Impact of PSFHI and Hospital Accreditation Centralized/District level monitoring for compliance of implementation of guidelines
**Infection, Prevention and Control**	PPE shortages and early overuse Weak IPC policies Poor implementation of IPC protocols Weak referral pathways Delays in testing Mismanagement of visitors Weak monitoring system on IPC compliance HRH KAP/Culture of negligence on IPC Weak knowledge and skills No adherence and misuse	IPC measures and trainings Daily PPE/supplies distribution Zoning and Altering infrastructure for IPC Limiting visitors Designating IPC officer Role of nurses Monitoring of IPC compliance

In responding to COVID-19 over the last year, hospitals have faced continuously changing challenges and have adapted to maintain operations, provide essential services, and minimize cross-infections of staff and patients. EMR hospitals responding to new variants of the virus and pressures of the pandemic continue to operate despite overwhelmed and exhausted staff, high levels of burnout, absenteeism, infection rates among hospital workers, excess numbers of severe and critical patients requiring longer than usual and resource-intensive hospitalizations causing shortages of ICU beds. Additionally, the shortages in life-saving drugs, inaccessible and costly medications, limited oxygen supply, implementation of non-evidence-based treatments, poor quality PPEs, and inadequate compliance with IPC measures pose complex challenges to hospitals operations and management. These challenges were found to be greater in the Region’s LMICs and exacerbated in FCS due to the economic and political pressures and additional health systems shocks affecting many EMR countries [32]. Moreso, hospitals continue to face challenges managing the pandemic in the face of the public disbelief in the virus, distrust in the efficacy of the vaccines, and negative perceptions of hospitals as places reserved for death. Nevertheless, the experiences of hospitals combatting COVID-19 in the EMR provide numerous insights on maintaining hospital operations to provide critical care and essential services, especially in FCS [97]. Hospitals in these settings implemented innovative solutions to treat critical patients, despite shortages of ICU beds, drugs, PPEs, qualified specialists, and consistent revenues. In line with findings from other resource-limited settings, EMR hospitals in humanitarian settings highlight the importance of operationalizing and implementing preparedness, agility, and resilience at the facility-level [31, 98].

Furthermore, strengthening hospitals’ preparedness through early, proactive, holistic and systems approaches is necessary not only in the face of subsequent waves of the pandemic, but essential to protecting national, regional, and global health security, and achieving universal health coverage [32]. In preparing hospitals for subsequent surges and planning for recovery, this study further highlighted the need for continuous evaluation and learning from these multi-pronged interventions across the various hospitals’ readiness domains. Improving EMR hospitals readiness, safety, and performance requires investment in preparedness, capacity building of hospital workers and managers on leadership, communication, HR management and IPC. Additionally, improvements to the quality of hospital services are needed through establishing appropriate and functional triage, testing and treatment, monitoring the implementation of clinical guidelines, and optimizing telemedicine to manage surges, maintain services, and strengthen capacities. Moreso, the findings of this study confirmed the need to strengthen hospitals’ resilience through a holistic and integrated health systems strengthening approach, especially in FCS [73, 99]. In these settings, innovative approaches are needed to integrate services delivery between all public, private, and humanitarian actors, train and retain health workers which are fit for emergency and outbreak response, improve supply chain mechanisms, and standardize guidelines to avoid fragmentation, duplication, and inefficiencies. With continuously evolving challenges, adaptability, and innovation in hospitals’ readiness for emergencies is necessary, increasing flexible expenditures for health at national and facility-levels along with hospitals autonomy with the appropriate accountability structures will facilitate decision-making and improve operations [97]. Developing leadership competencies of HMs, integrating public health expertise in hospital management structures, and strengthening health information systems and research capacities at facility-levels are essential learnings from EMR hospitals’ experiences combatting COVID-19 [100]. Furthermore, in building health systems resilience, multi-pronged (across each of the hospitals readiness domains) and multi-level policies are required to strengthen hospitals’ preparedness, response, and recovery throughout the various stages of the pandemic cycle [32].

One of the major strengths of this study is that it is among the first to capture hospitals experiences combatting the pandemic at a regional level. This study also addresses a gap in regional body of evidence by providing insights of the early experiences of hospitals’ preparedness and response, their continuously evolving challenges, and the interventions utilized to address them. These experiences provide invaluable lessons for policymakers and HMs across the Region, and context-specific insights for other LMICs, as the pandemic continues to unfold. On the other hand, this study was limited by the high workload, pressures, and limited time of HMs and short study period which constrained the number of KIIs and survey respondents. In some countries, only one or two key informants were interviewed, which does not sufficiently capture the experiences of hospitals throughout the entire country, while in others, KIs were HMs of larger tertiary hospitals whose experiences do not reflect those of peripheral hospitals in districts. As the pandemic continues to unfold and new evidence continues to be generated, newer publications and reports may have been missed. The changing challenges and interventions applied by hospitals in the early months of the response, compared to subsequent waves one year later, indicate that continuous and further documentation of experiences and lessons learned is needed, especially across the EMR’s LMICs and FCS. Moreover, as the pandemic continues with surges around religious holidays, this study revealed that hospitals play a key role in providing safe and timely critical care, rebuilding public trust in health systems, and providing reliable information on vaccines and preventive measures, issues that require further investigation. Additional research is also needed with regards to the early experiences and challenges of hospitals, evaluations of the impacts of various interventions (related to staff mental health and attitudes towards the pandemic, IPC, surge, and clinical management) and the cost effectiveness of these interventions towards building resilient hospitals for emergencies and outbreak response.

## Conclusion

In conclusion, this study highlighted the adaptability of EMR hospitals in addressing complex challenges to maintain operations, respond to emergencies, protect patients and staff, while also continuously evolving to strengthen their readiness for subsequent surges and plan for recovery. Policies and actions need to be implemented to ensure high-quality person-centered service delivery continues across the Region, with an emphasis on strengthening hospitals care and management as the backbone of this COVID-19 response. Furthermore, to achieve universal health coverage and protect global health security, we must prioritize rebuilding resilient health systems that are able to resist, absorb, accommodate, and recover from external shocks in a timely and efficient manner. Strengthening hospitals’ emergency preparedness and response is necessary in preparing health systems for future outbreaks and shocks.

## Supporting information

S1 Appendix Key informants’ information. (DOCX)
