# Supplementary material for: Hospitals early challenges and interventions combatting COVID-19 in the Eastern Mediterranean Region
Source: PLoS One. 2022 Jun 3;17(6):e0268386. doi: 10.1371/journal.pone.0268386 (PMC9165776; doi:10.1371/journal.pone.0268386)
Supplement: S1 Appendix — (DOCX) [file pone.0268386.s001.docx]

**S1 Annex: Key informants’ information**

| KI # | Country | KI Position/Role | KI Affiliation or Hospital Designation |
| --- | --- | --- | --- |
| 1 | Afghanistan | WHO Staff | Technical Officer |
| 3 | Bahrain | Hospital manager | Sulaymenia Medical Complex |
| 5 | Iraq | WHO Staff | Technical Officer |
| 8 | Iraq | Senior management team | District Hospital of Sulaimanyiah |
| 9 | Jordan | Senior management team | Deputy CEO |
| 14 | Oman | WHO Staff | Technical Officer |
| 15 | Oman | Hospital manager | Khoula Hospital |
| 16 | Oman | Hospital manager | Nizwa Hospital |
| 18 | Palestine | WHO Staff | Technical Officer |
| 19 | Palestine | Senior management team | Head of Infectious Diseases |
| 22 | Pakistan | Hospital manager | Indus Hospital Network |
| 26 | Pakistan | Senior management team | Agha Khan University Hospital |
| 29 | Somalia | Policymaker | Federal Ministry of Health |
| 30 | Somalia | Hospital manager | DeMartino Hospital |
| 34 | Sudan | Senior management team | University isolation center |
| 43 | Yemen | Senior management team | Al-Rawdha Hospital in Taiz |
